# Neural mechanism underlying preview effects and masked priming effects in visual word processing

**DOI:** 10.3758/s13414-024-02904-8

**Published:** 2024-07-02

**Authors:** Xin Huang, Brian W. L. Wong, Hezul Tin-Yan Ng, Werner Sommer, Olaf Dimigen, Urs Maurer

**Affiliations:** 1 Department of Psychology, The Chinese University of Hong Kong, Sino Building 3/F, Shatin, New Territories, Hong Kong, SAR China; 2 Institut für Psychologie, Humboldt-Universität zu Berlin, Berlin, Germany; 3 Department of Psychology, Zhejiang Normal University, Jin Hua, China; 4 Department of Psychology, University of Groningen, Grote Kruisstraat 2-1, 9712 TS Groningen, The Netherlands; 5 Centre for Developmental Psychology, The Chinese University of Hong Kong, Hong Kong, China; 6 Brain and Mind Institute, The Chinese University of Hong Kong, Hong Kong, China; 7 BCBL, Basque Center on Brain, Language and Cognition, Donostia-San Sebastián, Spain; 8 Wofoo Joseph Lee Consulting and Counselling Psychology Research Centre, Lingnan University, Hong Kong, China; 9 The Research School of Behavioural and Cognitive Neurosciences, University of Groningen, Groningen, the Netherlands; 10 Department of Physics and Life Science Imaging Centre, Hong Kong Baptist University, Hong Kong, China

**Keywords:** Single word boundary paradigm, Masked priming, N1, N250, Preview effect

## Abstract

Two classic experimental paradigms – masked repetition priming and the boundary paradigm – have played a pivotal role in understanding the process of visual word recognition. Traditionally, these paradigms have been employed by different communities of researchers, with their own long-standing research traditions. Nevertheless, a review of the literature suggests that the brain-electric correlates of word processing established with both paradigms may show interesting similarities, in particular with regard to the location, timing, and direction of N1 and N250 effects. However, as of yet, no direct comparison has been undertaken between the two paradigms. In the current study, we used combined eye-tracking/EEG to perform such a within-subject comparison using the same materials (single Chinese characters) as stimuli. To facilitate direct comparisons, we used a simplified version of the boundary paradigm – the single word boundary paradigm. Our results show the typical early repetition effects of N1 and N250 for both paradigms. However, repetition effects in N250 (i.e., a reduced negativity following identical-word primes/previews as compared to different-word primes/previews) were larger with the single word boundary paradigm than with masked priming. For N1 effects, repetition effects were similar across the two paradigms, showing a larger N1 after repetitions as compared to alternations. Therefore, the results indicate that at the neural level, a briefly presented and masked foveal prime produces qualitatively similar facilitatory effects on visual word recognition as a parafoveal preview before a single saccade, although such effects appear to be stronger in the latter case.

**Supplementary Information:**

The online version contains supplementary material available at 10.3758/s13414-024-02904-8.

## Introduction

In research on reading, two classic experimental approaches – masked repetition priming (Forster et al., 1987) and the boundary paradigm (Rayner, 1975) – have played an important role in understanding the facilitatory effects of foveal and parafoveal information on word recognition. In the masked priming paradigm, a target word is preceded either by the same word or by an unrelated word as a briefly presented foveal prime. In a typical experiment using the boundary paradigm, readers fixate target words for which either the same word or an unrelated preview had been shown in parafoveal vision during the preceding fixation. The preview is defined as the stimulus presented parafoveally at a location before that location is fixated. The facilitatory effects observed with both paradigms have been used to develop and refine models of reading; for example, the bi-modal interactive-activation model (Grainger & Holcomb, 2010) can explain masked priming effects, whereas the E-Z reader model (Reichle et al., 2006) can account for many of the effects of parafoveal previews on fixation times in reading. Yet, the results of both paradigms have been rarely directly related to each other. Whereas masked priming studies have usually presented only individual words or word pairs, boundary studies have most commonly presented full sentences. Furthermore, behavioural studies with both paradigms have typically used different dependent variables (reaction times in the masked priming paradigm and fixation durations in the boundary paradigm), making the effects difficult to compare. In contrast, electrophysiological recordings should allow for direct comparison between the facilitatory effects in paradigms. In the following, we briefly discuss both paradigms and then summarize the current study, which compared the effects of foveal and parafoveal facilitation in both paradigms directly within the same participants.

### Masked repetition priming paradigm

In a typical masked priming paradigm, a foveal prime is presented for a brief duration (i.e., 40–72 ms) followed by a mask stimulus, which causes backward masking of the prime, making it consciously imperceptible. Behavioural responses (e.g., reaction times) to a subsequently presented target word are facilitated, if the target word is preceded by an identical word, even if there is a case change between prime and target (e.g., Forster et al., 1987; Forster & Veres, 1998). More recently, studies using event-related potentials (ERPs) have investigated the neural mechanisms involved in masked priming (Grainger et al., 2012; Holcomb et al., 2005; Holcomb & Grainger, 2006, 2007; Kiefer & Brendel, 2006). In alphabetic languages (masked) repetition of visual words typically led to a reduction in two ERP components compared to unrelated words, the N250 and the N400 components (Chauncey et al., 2008; Holcomb & Grainger, 2006). The N250 repetition effect has a scalp distribution with the largest activation over midline and slightly anterior left hemisphere sites if it is recorded with a mastoid reference. At occipital electrodes, the effect shows a distribution with inverted polarity with a reduced negativity following repetitions, if an average reference is used (Huang et al., 2022). The N250 repetition effect seems to be sensitive to the visual-orthographic properties of the words, as it can be enhanced when primes and targets show orthographic overlap. In contrast, the N400 effect may reflect post-lexical semantic processing (see Kutas & Federmeier, 2011, for a review). In masked repetition priming studies, the N400 repetition effects shows a right-hemispheric lateralization (Chauncey et al., 2008; Holcomb & Grainger, 2006), with the largest activation over central-posterior scalp sites. The reduction of the N250 and N400 components may be considered as a correlate of the facilitation of orthographic and semantic processes (Grainger et al., 2012; Holcomb & Grainger, 2006).

In addition to the N250 and N400 effects, some studies also found repetition effects in the N1 component at occipital electrodes with an increased negativity for same-word versus different-word primes (Chauncey et al., 2008; Huang et al., 2022). This N1 component has also been called the N/P150 component, as it is a highly focal bipolar ERP effect, with a positivity polarity at occipital scalp sites and a negativity polarity at anterior sites. Compared with the N250 and N400 effects, the N1 effect seems to be less consistent and less robust, possibly due to the use of mastoid references, a reference montage that is close to the electrodes where the N1 is maximal (Huang et al., 2022). In masked priming experiments, the early N1 component has been found to be not only sensitive to linguistic stimuli, like words (Holcomb & Grainger, 2006) and single letters (Petit et al., 2006), but also to non-linguistic stimuli, like pictures of objects (Eddy et al., 2007), suggesting that the N1 may reflect an early process involved in mapping visual features onto higher-level representations (Grainger & Holcomb, 2010).

Masked repetition priming studies in Chinese showed a similar pattern to alphabetic languages, that is, reduced N250 (Huang et al., 2022; Wong et al., 2014) and N400 amplitudes (Du et al., 2013; Wong et al., 2014; Zhang et al., 2012). These studies show that masked priming effects in N250 and N400 components are commonly obtained in visual word recognition irrespective of the properties of the writing system; hence, the N250 repetition effect might be a good candidate to compare facilitation effects at the level of early visual orthographic processing across different writing systems.

### Boundary paradigm

In experiments using the boundary paradigm (Rayner, 1975), also known as the gaze-contingent boundary paradigm, readers read words or sentences normally with eye movements. When their eyes move from a pre-target word *n* to a target word *n* + 1, that is, once the eye gaze crosses an invisible boundary located in-between the words, the target word *n* + 1 is either replaced by another word or it remains the same word that was previewed in parafoveal vision during fixations on word *n*. In other words, fixations of word *n* + 1 occur either after a valid or after an invalid parafoveal preview of the word. The key finding in this paradigm is that fixation durations after valid previews are shorter than after invalid previews (the preview effect), suggesting that the response to a valid preview is facilitated compared to invalid previews (for a review, see Hyönä, 2012; Schotter et al., 2012a; Vasilev & Angele, 2017a).

At the electrophysiological level, studies combining EEG and eye-tracking have found a reduced negativity in fixation-related potentials (FRPs) following fixations on word *n*+1 if the preview was valid as compared to invalid (Dimigen et al., 2012; Kornrumpf et al., 2016). This effect, which has also been called “preview positivity”, is largest in a time window between 200 and 280 ms after fixating word *n* + 1 and maximal over occipital-temporal scalp sites. In subsequent studies, the preview positivity was not only observed in word list reading (Dimigen et al., 2012), but has also been replicated in reading natural sentences (e.g., Antúnez et al., 2022; Degno et al., 2019a, 2019b; Dimigen & Ehinger, 2021; Li et al., 2024) as well as in studies using the RSVP-with-flanker-word presentation approach in Chinese readers (Li et al., 2015, 2022, 2024). While this effect has often been referred to a “late N1 effect”, its time window and occipito-temporal scalp distribution are similar to those of the N250 component observed in masked priming studies (e.g., Holcomb & Grainger, 2007). Besides the preview positivity, a reduced N400 negativity to valid as compared to invalid preview has been observed between 300 and 500 ms after fixation onset (Dimigen et al., 2012; Li et al., 2022), although not all studies show this effect (Degno et al., 2019b). In word list reading, the N400 effects were only obtained with identical previews, whereas semantically associated previews did not facilitate the process (Dimigen et al., 2012). However, in more natural reading setting (i.e., sentences), both identical and semantic associations seem to affect the fixation-related brain response (Antúnez et al., 2022; Li et al., 2022, 2024), indicating that sentence meaning rapidly adapts to the parafoveal preview.

Compared to the preview positivity and N400 effect, the early part of the N1 effect was less investigated or reported in previous studies (Dimigen et al., 2012; Li et al., 2015; Niefind & Dimigen, 2016). Degno et al. (2019a) identified two distinct clusters within the 120- to 300-ms time window for all main effects and interactions, which were suggested to reflect to the N/P150 and N250 components. This N1 effect has a scalp distribution with the largest activation in the occipito-temporal regions, being more negative for valid than unrelated previews. According to the visual inspection on the waveforms of the published studies (Dimigen et al., 2012), compared to the preview positivity, the N1 effect is usually smaller and less robust, and also less consistent.

### Similarities and differences between masked priming and the boundary paradigm

Of course, the two paradigms differ in several important ways, including the stimulation itself. First, the locations of primes/previews are different. As mentioned earlier, primes in masked repetition priming studies are usually passively presented in the screen center (fovea), where participants are fixating while the words are shown. In the boundary paradigms, participants need to move their eyes actively and previews usually appear in the parafovea of the right visual field, which projects information to the left hemisphere. This means that there is a difference in attention orientation or attention allocation between the paradigms. A previous study comparing foveal-in-fovea priming to parafovea-on-fovea priming showed that foveal priming effects were bilateral whereas parafoveal priming effects were left-lateralized (Pernet et al., 2007), indicating that the locations of primes influence the neural processes. Therefore, it is possible that the foveal masked priming and parafoveal preview effect have different neural processes.

Second, in a masked repetition priming paradigm, the primes are usually masked with a backward mask (e.g., hash mark), which also causes a delay between primes and targets. In contrast, with the boundary paradigm, the preview is typically immediately replaced by the target, although this happens during the saccade towards the word (while there is strong saccade-related motion smear on the retina; Schweitzer et al., 2023), which will mask some high spatial-frequency information. Therefore, it raises another question about whether the existence of a delay and mask between prime/preview and target influences word recognition differently.

Finally, whereas the masked priming paradigm only uses single words or word pairs (prime and target), the previews in studies using the boundary paradigm are most commonly embedded within full sentences. This means that context-based predictions affect the processing of the preview and target in the latter paradigm. However, preview effects in fixation times and in fixation-related brain activity are also reliably observed with simplified variants of the boundary paradigm, where the reader only reads short, context-free lists of unrelated nouns (Dimigen et al., 2012; Kornrumpf et al., 2016; Niefind & Dimigen, 2016).

Interestingly, based on a literature review, the N250 repetition effects appear to be similar in terms of time window and scalp distribution between masked priming and boundary paradigm. They both showed largest activation over occipito-temporal sites within a time window of around 180–280 ms. Previous studies (e.g., Dimigen et al., 2012; Holcomb & Grainger, 2006) have found that valid primes/previews could facilitate the neural process compared to invalid primes/previews, as reflected in a reduced N250 negativity for valid primes/previews compared to invalid primes/previews.

In terms of N400 effects, the two paradigms also seemingly show some similarities. In the time window of 300–500 ms, valid primes/previews elicited smaller amplitudes than invalid primes/previews. In masked priming studies, N400 effects usually have a posterior and left-lateralized maximum in an early phase but a more posterior and right-lateralized maximum at a later phase (Holcomb et al., 2005; Kiyonaga et al., 2007). In the boundary paradigm, the N400 effects usually have a centroparietal scalp distribution (as in masked priming studies), but they are less consistent, with highly robust N400 effects mainly found in RSVP-with-flanker paradigms (e.g., Li et al., 2015, 2022). In FRP studies, the evidence is more mixed; here, marginally significant effects (e.g., Dimigen et al., 2012), polarity-reversed effects (Degno et al., 2019a), but also robust N400 effects (Mirault et al., 2020; Li et al., 2024) have been reported.

With regard to the N1 component, a review of published figures suggests that the scalp distribution and direction of effects may be similar between masked priming and the boundary paradigm. However, given the fact that the early N1 repetition effect was rarely formally reported in previous boundary paradigm studies, it is not known whether the similarities generalize to all studies. The N1 repetition consists of an increased negativity for same versus different word presentations at occipital sites (Chauncey et al., 2008; Huang et al., 2022; Kornrumpf et al., 2016).

In terms of behavior measures, in masked priming studies, although the reaction times for repetition effects were not always reported (e.g., Chauncey et al., 2008; Holcomb & Grainger, 2007), the valid primes usually lead to shorter reaction times than unrelated primes (Morris et al., 2011; Morris & Stockall, 2012). In contrast, in boundary paradigms, fixation durations are usually analyzed, and it has been found that valid previews usually lead to 20- to 30-ms shorter fixation durations (for a review, see Vasilev & Angele, 2017a).

Although the early N1 and N250 effects show apparent similarities, it is unclear whether the early effects elicited in the two paradigms are actually the same. It is not known whether the same effects in terms of ERP amplitudes, topographies, and latencies are found if the same materials are presented in both paradigms. To establish better links between the literatures on both effects, it is important to understand the similarities and differences between the two paradigms using EEG measures. In the current study, for the first time, we used the same materials to investigate the neural correlates underlying the masked priming and boundary paradigm recorded in the same participants. We hope that this will also allow us and others to span a bridge between these two important lines of research and to cross-reference the results and their theoretical interpretations in the respective literatures. A direct comparison of the two paradigms may also allow us to understand the EEG components better, as previous discussions and functional interpretations typically only occurred within the framework of one paradigm (e.g., Dimigen et al., 2012; Grainger & Holcomb, 2009). Grainger et al. (2016) conducted an indirect comparison between central priming and parafoveal priming. In their study, they assessed repetition priming effects by presenting targets both centrally and parafoveally, with primes always presented centrally. Their findings revealed that repetition effects were delayed when the targets were located in the peripheral vision. While trans-saccadic repetition priming differs from the boundary preview effect, this research offers direct evidence regarding the influence of attention allocation speed to the periphery and the quality of information available in the peripheral vision prior to target stimuli fixation. The study also demonstrated that a forward mask could facilitate the shift of attention to the target location before the target's onset. Consequently, this research suggests that when comparing the masked priming paradigm with the boundary paradigm, both spatial integration and the quality of previews/primes can significantly impact repetition effects.

In addressing the cognitive processes underlying reading, a notable distinction emerges between single word reading and sentence reading paradigms. This paper aims to explore this dichotomy, and may further motivate the study of using word lists and connected sentences. Such an approach allows for an in-depth examination of the degree to which preview and masked priming effects are analogous. This distinction is vital to understand the inherent methodological confound arising from contrasting the boundary paradigm, which focuses on sentence reading, with the masked priming paradigm that involves the use of isolated words.

Our exploration is guided by a pivotal question: whether the neural phenomena associated with repetition effects in both paradigms are similar or different. By investigating this, we aim to infer whether similar mechanisms are at play or if the paradigms involve distinct mechanisms. This comparative analysis is essential, as it helps to identify the unique and overlapping aspects of cognitive processing in different reading formats.

Historically, eye-tracking studies and boundary paradigms were developed to investigate naturalistic reading behaviors. In contrast, EEG studies probing neural mechanisms of reading were constrained to more artificial paradigms, primarily due to the complications posed by eye-movement artifacts. With the advent of advanced techniques that facilitate the co-registration of EEG and eye tracking, along with the ability to correct for these artifacts, it is now feasible to study the neural mechanisms of reading in settings that more closely resemble natural reading conditions.

Given the extensive insights garnered from single word processing studies, it becomes imperative to juxtapose these findings with those obtained from more naturalistic paradigms. This study, therefore, represents an essential step towards integrating more realistic elements into ERP reading research, as we incorporate an eye movement into our paradigm. This approach marks a transition towards investigating preview effects in sentence reading and also aligns with the recommendations of our reviewers. By bridging the gap between artificial paradigms and naturalistic reading conditions, our study aims to enhance the understanding of the neural underpinnings of reading, thereby contributing a significant perspective to the existing body of literature in this field.

### Current study

Given what appears to be considerable similarities of ERP effects in masked priming and boundary paradigm studies on visual word recognition, it seems important to directly compare these effects in the same study. Demonstrating a similarity of the neural facilitation effects that have long but separate traditions in research on visual word processing would allow researchers to compare results better and link theoretical interpretations between the two lines of research. Therefore, in the current study, we compared neural facilitation effects between masked priming and boundary paradigms in the same participants, with the same stimuli, as well as the same EEG setup and pre-processing steps. We also used simultaneous eye tracking to co-register eye movements with the EEG. As mentioned in the previous section, the traditional boundary paradigm has most commonly been used to study normal reading and is commonly employed in scenarios in which readers read full sentences. In contrast, the masked priming paradigm is designed to examine the processing of isolated visual words. Consequently, we adopted the paradigm proposed by Rayner et al. (1980), which is specifically geared towards single-word assessments. To distinguish this paradigm from more typical use of the boundary paradigm with sentences, we refer to this approach here simply as the *single word boundary paradigm*. This terminology allows us to contrast the neural processes underpinning foveal and parafoveal repetitions for visual word recognition, and to emphasize that our results may differ in some aspects from those obtained with the classic boundary paradigm during sentence reading, where the preview effects are also modulated by contextual predictability. For the purposes of this study, we refer to the character presented in the parafovea as the preview. This term is usually used for the parafoveal stimulus presented during gaze-contingent display change experiments in sentence reading, but we feel the manipulation we used is similar enough to warrant using this term.

For the EEG measures, we focused on three components: the N1, N250, and N400 component. While the N250 and N400 effects are consistently observed across the two paradigms, the N1 effect appears less robust. We included the N1 for the following reasons. Our previous studies using different tasks with Chinese characters as stimuli consistently showed robust N1 effects (Huang et al., 2022; Maurer et al., 2023). This N1 effect appears to be consistent across various tasks and paradigms, including the masked priming paradigm (Huang et al., 2022). Hence, we expected to observe N1 effects in both paradigms used in the current study. The variability of the N1 effects across studies might be attributed to the stimuli. Since the N1 component is sensitive to the visual feature mapping, the effects tend to be more pronounced when the primes are physically distinct from the targets. Given that Chinese characters are visually more complex than English words, we expected that the N1 effects in the present study would be larger compared to previous studies.

All hypotheses and analyses were pre-registered on the Open Science Framework at https://osf.io/hk37q/. Behaviourally, we expected to replicate the typical preview effects in terms of fixation durations in the single word boundary paradigm, although most studies obtained the preview benefit on eye movements through natural sentence reading (e.g., Yan et al., 2009; Yang et al., 2012) rather than during single character reading. Electrophysiologically, we expected that both paradigms would show an attenuated (less negative) N250 in response to a stimulus following an identical prime/preview as compared to an unrelated prime/preview. This effect was expected to be larger for the single word boundary paradigm compared to the masked priming experiment based on previous studies (Dimigen et al., 2012; Petit et al., 2006), as active oculomotor behaviour seems to modulate word recognition under otherwise comparable conditions (Kornrumpf et al., 2016). With regard to the N1 component, the boundary paradigm was expected to result in an increased (more negative) early N1 after identical compared to unrelated previews. This effect was expected to be larger than in the masked priming experiment (i.e., interaction with factor *Paradigm*). The interaction might result from a reduced/absent effect in the masked priming experiment or even from an effect in the masked priming paradigm that reverses direction (larger negativity after related than unrelated stimuli; Grainger et al., 2012). For our analysis, we also used a time-point-to-time-point Topographic Analysis of Variance (TANOVA) that includes all electrodes to test at which time points the masked priming effect and preview effect would occur across all electrodes. Although not pre-registered, at the request of the reviewers, the N400 effects were also included to examine the differences and similarities of the two paradigms.

## Methods

### Participants

Twenty-nine native Cantonese-speaking Chinese participants (13 males; mean age = 19.21 years, range = 18–25 years) were tested in both paradigms. Data from one additional participant were excluded from the analysis due to a small number of trials performed (average trial number in each condition < 15). All participants had normal or corrected-to-normal vision, with no history of dyslexia or ADHD (self-report). They were all right-handed as determined by the Chinese Handedness Questionnaire (Li, 1983). Written informed consent was obtained prior to the experiment. All participants were reimbursed 50 Hong Kong dollars (about 7 USD) per hour. The study was approved by the Joint Chinese University of Hong Kong‐New Territories East Cluster Clinical Research Ethics Committee.

### Materials

Eighty-four traditional Chinese characters were selected from the Chinese Character Database (Kwan et al., 2012) as target characters. These 84 characters were paired with either identical or unrelated characters, therefore, a total of 168 trials were included in the data analysis. The unrelated characters and target characters were matched according to stroke numbers (*t*_(166)_ = 0.04, *p* = 0.97), radical family size (*t*_(166)_ = 0.35, *p* = 0.73) and frequency (*t*_(166)_ = 0.07, *p* = 0.95). There were no shared radicals and homophones within a pair.

An additional 16 animal characters served as probes and were either presented in the prime/preview or target position with equal probability (4%). Each animal character was paired once with an unrelated character and once with the identical animal character, resulting in 16 trials serving as the unrelated condition, and 16 trials serving as the repeated condition. Of the 16 trials serving as the unrelated condition, eight trials had the animal character presented in the preview/prime position, and eight trials had the animal characters presented in the target position. As a result, participants saw each target character four times. In total, there were 100 pairs of trials with unrelated preview/prime and 100 pairs with an identical preview/prime. For trials containing animal names, participants were instructed to press a button (response hand counterbalanced across participants) whenever they detected an animal name in either the prime or the target position. Trials with animal characters were excluded from the EEG data analysis. With this design, the stimuli used in the two paradigms were the same, including the characters indicating animacy. The order of items within each paradigm was randomized. The order of the two paradigms was counterbalanced across participants.

The animal names served as probe items in a semantic categorization task in which participants were instructed to rapidly press a single button whenever they detected an animal name in either the prime/preview or target position. A practice session was administered before the experiment to familiarize the participant with the procedure.

### Procedure

#### Masked priming paradigm

**Fig. 1:**
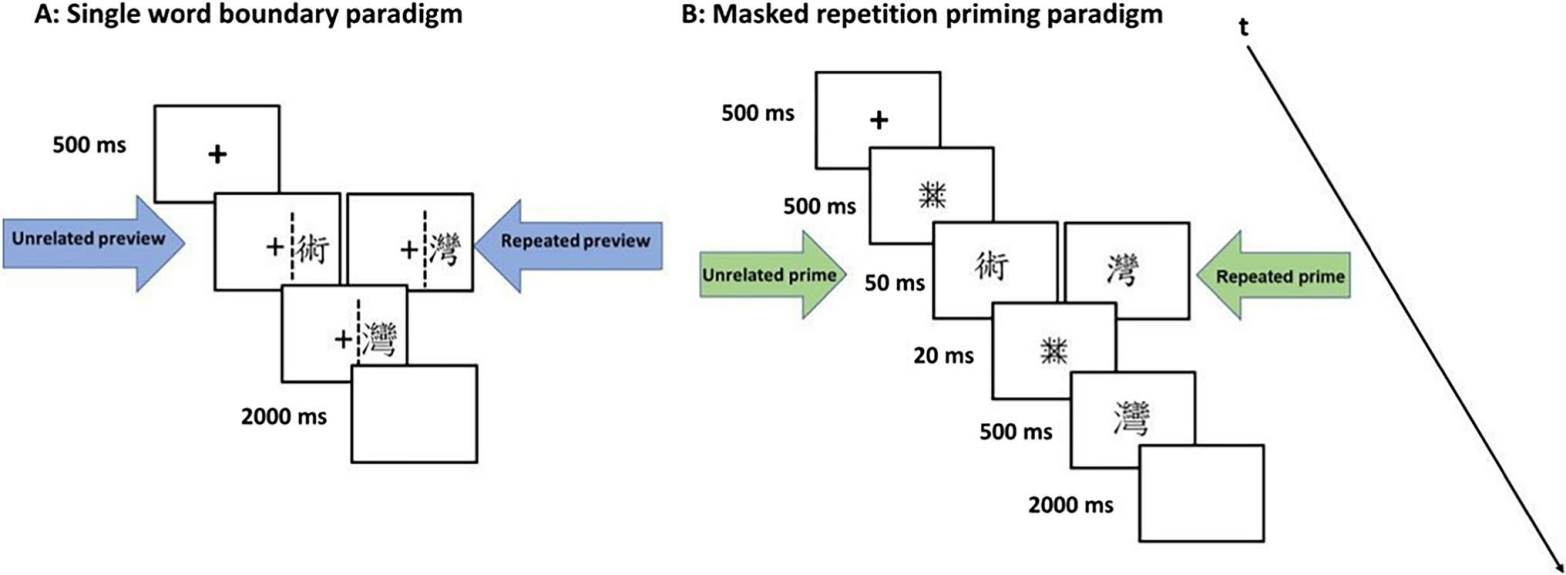
Schematic representation of a typical trial in the single word boundary paradigm (**A**) and the masked priming (**B**). The dashed line in the single word boundary paradigm indicates the invisible vertical boundary. Once the eyes cross this boundary, during the saccade, the preview is exchanged to the target. The figure shows an example of a trial in which the prime/preview was unrelated (left) and repeated/valid (right) to the target character, respectively. In this example, “術” means technique, “灣” means bay. All stimuli were presented in black on a white background

Visual stimuli were presented on a 24-in. monitor (BenQ ZOWIE XL2411K, resolution: 1,920 × 1,080 pixels; vertical refresh rate: 144 Hz) and located at a distance of 90 cm directly in front of the participant. Stimuli were displayed in high contrast as black characters on a white background. As shown in Fig. 1B, each trial began with a fixation cross in the middle of the screen for 500 ms, followed by a forward mask with a duration of 500 ms. The forward mask was replaced at the same location on the screen by a prime character (set in a Kaiti font) for 50 ms. The prime was then immediately replaced by a backward mask. The backward mask remained on-screen for 20 ms and was immediately replaced by the visual target (in the PMingLiu font) for a duration of 500 ms. Target word presentation was followed by a 2,000-ms long empty white screen. When the target disappeared, marking the end of the trial, participants were asked to press the button to indicate whether they had seen an animal name during the trial or not (regardless of the position of this probe as prime or target). In the masked priming paradigm, participants were asked to refrain from blinking and from moving their eyes while stimuli were presented.

#### Single word boundary paradigm

Technical settings were the same as in the masked priming paradigm except for the schematic of the trial (see Fig. 1A for a schematic of a typical trial). In the single word boundary paradigm, each trial also started with a fixation cross in the middle of the screen. Participants needed to fixate on this cross within 3 s (fixation check). Afterwards, the preview character appeared centered at a visual angle of 5.5° to the right of fixation, and participants moved their eyes rightwards towards it. The distance from the fixation cross to the left edge of the character was 3.03°. An invisible boundary was located in-between fixation cross and character, at an eccentricity of 2.5° to the right of fixation. The trial was aborted if the boundary was not crossed after 3,000 ms. As with the masked priming experiment, the preview character was presented in Kaiti font. Once the participants’ eyes moved across the invisible boundary, the parafoveal character was replaced by the target character in a PMingLiu font. Following the crossing of the invisible boundary, targets were presented for a duration of 1,000 ms.^1^ As in the masked priming experiment, once the target disappeared, there was a 2,000-ms blank white screen and participants were then asked to press the button to indicate whether they had seen an animal name, without being informed of the position where the name would appear (again, regardless of the position of this probe as preview or target).

Display change awareness was assessed in a structured interview after both experiments. Participants were first asked whether they had noticed “anything strange about the visual display of the text” (White et al., 2005). If they answered “no”, they were informed that changes had taken place and asked again whether they had noticed any. If they did, participants were asked to estimate the number of changes perceived. Due to the simplified single-saccade setting of the current reading task, we expected that most participants would be aware of the display changes (from parafoveal character to target) in the single word boundary paradigm trials. This was also the case for the masked priming paradigm as there were visual transients on the display. Notably, however, N1 effects in the boundary paradigm have been shown to arise regardless of display change awareness (Dimigen et al., 2012).

### EEG recordings

The EEG was recorded from 64 Ag/AgCl scalp electrodes mounted in a textile cap at standard 10–10 positions and referenced online against CPz. Two electro-oculogram (EOG) electrodes were placed on the outer canthus of each eye and one EOG was placed on the infraorbital ridge of the left eye. Signals were amplified with an EEGO amplifier system (Advanced Neuro Technology, Enschede, Netherlands) at a bandpass of 0.01–70 Hz and sampled at 1,000 Hz. Impedances were kept below 20 kΩ.

### Eye-movement recordings

The eye movements were recorded binocularly through a desktop-mounted Eyelink 1000 Plus eye-tracking system (SR Research) at a sampling rate of 1,000 Hz. The head position was stabilized via the chin rest of the tracker. A 9-point calibration was completed at the beginning of each paradigm. In addition, a 1-point drift correction check was performed at the beginning of each trial. Extra calibrations were performed whenever a fixation check failed. A calibration was accepted if the average validation error was below 0.5° and the maximum error across all points was below 1.0°.

### Co-registration of eye movements and EEG signal

Synchronization of eye movements and EEG was achieved by sending shared trigger pulses from the presentation PC (running Presentation, Neurobehavioral Systems Inc., Albany, CA) to the EEG and eye-tracking computer on each trial through the parallel port. This allowed for accurate offline synchronization via the EYE-EEG extension for EEGLAB (http://www.eyetracking-eeg.org; Dimigen, 2020; Dimigen et al., 2011). Differences in the markers from both recordings were at most 2 ms in absolute value (M = 0.45, SD = 0.09), verifying the high quality of synchronization.

### Pre-processing of eye-movement data

We used first-fixation durations (FFDs, the duration of the first fixation on a word) for data analysis. The EyeLink Parser performed online eye-motion event detection and processing, including fixations and saccades, and recorded both raw gaze points and these results. Only fixations that occurred during the first-pass reading in correct trials were analysed. Specifically, fixations on the area of interest were excluded, when the display change occurred too early or too late (i.e., when the display change took more than 10 ms before/after fixation onset on the target character). We also removed FFDs < 60 ms or > 600 ms (total number of excluded fixations: 1,844) and we excluded fixations on target characters in which participants blinked. In addition, we removed all trials with a wrong manual response to the animal question. Finally, we removed trials in which the display change was already triggered before saccade onset (‘early’ eye movements, *n* = 1,482, 25.72%; see *Discussion*), as parafoveal stimuli were not presented in the parafoveal area. Out of total 4,836 observations in the single word boundary paradigm, we kept 3,561 observations for all participants.

### EEG pre-processing

Offline, EEG data were digitally band-pass filtered using the *eegfiltnew.m* function of EEGLAB 2020.0 (Delorme & Makeig, 2004) toolbox for Matlab (version 2018b), between 0.1 Hz and 30 Hz (-6 dB/octave) and re-referenced to the average reference (Lehmann & Skrandies, 1980). Independent component analysis (ICA) was performed in order to identify the ocular artefacts using the EYE-EEG extension. ICA components that covaried with eye movements (variance threshold 1.1; Dimigen, 2020; Plöchl et al., 2012) were considered ocular artefacts and removed from the data (single word boundary paradigm: *M* = 6.76, *range*: 3–12; masked priming paradigm: *M* = 5.55, *range*: 3–12). The corrected EEG signal was then segmented from 300 ms prior to and 700 ms after the first fixation onset on the target character and baseline-corrected by subtracting the voltages during the 200-ms interval preceding the fixation onset on the target word. Trials with amplitudes exceeding ±100 µV in any channel were automatically rejected from further analyses. ERPs were first averaged within and then across participants.

After eye-movement and EEG pre-processing, we were left with a total of 3,393 (out of 3,561) observations for the target character in the single word boundary paradigm and 4,699 (out of 4,802) observations in the masked priming experiments. In each paradigm, participants had similar numbers of trials between repeated and unrelated conditions (boundary: *M* = 58.5, *SD* = 14.59, *range* = 31–81; masked priming: *M* = 81.02, *SD* = 4.27, *range* = 62–84). However, the total trials remaining between the two paradigms was significantly different (*t*_(114)_ = −11.28, *p* < 0.001). The reason for this difference was that the single word boundary paradigm required more eye movements and therefore had more artefacts that could not be corrected compared with the masked priming paradigm (see Nikolaev et al., 2016, for discussion). Moreover, we excluded short fixations and “early” eye movements, which left fewer trials compared to the masked priming paradigm. Therefore, the two experiments were different in the degree of technical challenges, which resulted in a different number of EEG trials that could eventually be included.

### Data analysis

#### Eye-movement statistical analyses

Eye-movement data were analysed with linear mixed-effects models within the R environment for statistical computing (R Core Team, 2015). We used the “lmer” function from the *lme4* package (Bates et al., 2015) on log-transformed FFDs.

The within-subject factors of *Repetition* (identical vs. unrelated) and *Paradigm* (masked priming vs. single word boundary paradigm) were coded as fixed factors. Participants and items were specified as crossed random effects, with both random intercepts and random slopes (Barr et al., 2013). When we ran the models, we always began with full models that included the maximum random effects structure, but the slopes were removed if the model failed to converge (indicating over parametrization). The *p*-values were estimated using the “lmerTest” package, with the default Satterthwaites’s approximation for the degrees of freedom and *t*-statistics (Kuznetsova et al., 2017).

#### Pre-registered EEG/FRP data analysis(https://osf.io/hk37q/)

##### Single word boundary paradigm

To investigate the neural correlates of the repetition effects, we analysed FRP epochs time-locked to the fixation onset of the target. Based on previous studies, we selected 200–300 ms as the time window of preview positivity (Dimigen et al., 2012). In addition, as many studies observed the early N1 component, we selected 140–200 ms as the time window of N1. As the N1 effects and preview positivity have a scalp distribution on the occipital temporal regions with an average reference, we selected these areas as the regions of interest (ROIs; left occipital-temporal area, LOT: PO9/PO7, and right occipital-temporal area, ROT: PO8/PO10) with a factor of *Hemisphere* (left vs. right). Therefore, Repeated-Measure Analyses of Variance (ANOVAs, with Bonferroni correction) were performed on the type of previews (*Repetition*, repeated vs. unrelated) and *Hemisphere* (left vs. right) for the N1 and N250 components.

##### Masked priming paradigm

The data analyses of the masked priming paradigm were similar to those of the single word boundary paradigm, except for the selection of time windows. Based on the previous studies, the N1 effects occurred earlier than that of the single word boundary paradigm, therefore the time window of N1 in the masked priming paradigm was 120–175 ms. The N250 component had a similar time window to the preview positivity based on previous studies (Petit et al., 2006). The same time window for the N400 component from the single word boundary paradigm also applied to the masked priming paradigm.

##### Comparison between two paradigms

To further investigate whether the repetition effects were similar across the two paradigms, we also performed three-way ANOVAs by assessing the interaction between the types of preview/prime and types of paradigms. Factors included *Repetition* (repeated vs. unrelated), *Paradigm* (masked priming vs. boundary) and *Hemisphere* (left vs. right; except for the N400 component). Greenhouse-Geisser correction was applied to all effects with more than one degree of freedom in the numerator.

ANOVA analysis is commonly employed in masked priming and boundary paradigm studies. We opted to use ANOVA to ensure that our results were comparable to those of previous studies. In addition to ANOVA, we conducted a regression analysis on the FRPs data (see Online Supplementary Material (OSM) Table 1). The results from both methods were consistent, suggesting that our findings were not influenced by the choice of statistical method.

#### Exploratory EEG/ FRP data analysis

As proposed in the pre-registration, we also explored the results with sample-by-sample Topographic Analyses of Variance (TANOVA; Koenig et al., 2011) on non-normalized (raw) maps comparing target ERPs/FRPs following valid primes/previews to those following invalid primes for unchanged conditions. The TANOVA was corrected for multiple comparisons through Global Duration Statistics (Koenig et al., 2011). Based on the TANOVA results, we selected the time windows in which repetition effects were significant (*p* < 0.05). As we were mainly interested in repetition effects corresponding to the N1 and N250 effects, we focused on the effects occurring within 300 ms after the target was presented/foveated. TANOVA comparing repeated and unrelated targets identified two significant time windows in each paradigm within 300 ms.

#### Behavioural data analysis

Since manual responses were only executed following a 2,000-ms blank screen interval, we did not analyse manual RTs in this study. In contrast, we calculated d-prime (*d’*) separately for probes in the preview/prime position and in the target position. This allowed us to quantify the visibility of the prime (masked priming) and the parafoveal preview (single word boundary paradigm) in the respective paradigm. Here, we call this ability to identify the prime/preview word *prime visibility*. In addition, we assessed the display change awareness for both paradigms.

## Results

### Preview effect in fixation times (Single word boundary paradigm)

To keep our single word boundary paradigm condition as comparable as possible to the masked priming condition, it only involved a single saccade. Following this saccade, the readers’ eyes remained fixated on the target word until the end of the trial. In this regard, our design resembled that of other recent EEG preview experiments with a single saccade (e.g., Buonocore et al., 2020; De Lissa et al., 2019; Huber-Huber et al., 2019). Due to the absence of an outgoing saccade going away from the target character, we did not expect to find the typical preview benefit in fixation times. In fact, the only measure that can be sensibly computed in our case is the duration of the first fixation (FFD), which is defined as the interval until the first refixation saccade on the target. This FFD on the target character did not differ between conditions (repeated: *M* = 341 ms, *SE* = 25; unrelated: *M* = 328 ms, *SE* = 25;* β* = −0.04, *SE* = 0.02, *t* = −1.59, *p* = 0.11). Single fixation durations and gaze durations on the target character were effectively terminated by the end of the trial and therefore not analysed. In summary, we did not observe preview benefits in fixation times, but our paradigm was also not optimized for this purpose.

### EEG results

All EEG results mentioned below were from pre-registered analyses, unless otherwise indicated.

#### Traditional ANOVA results

**Fig. 2:**
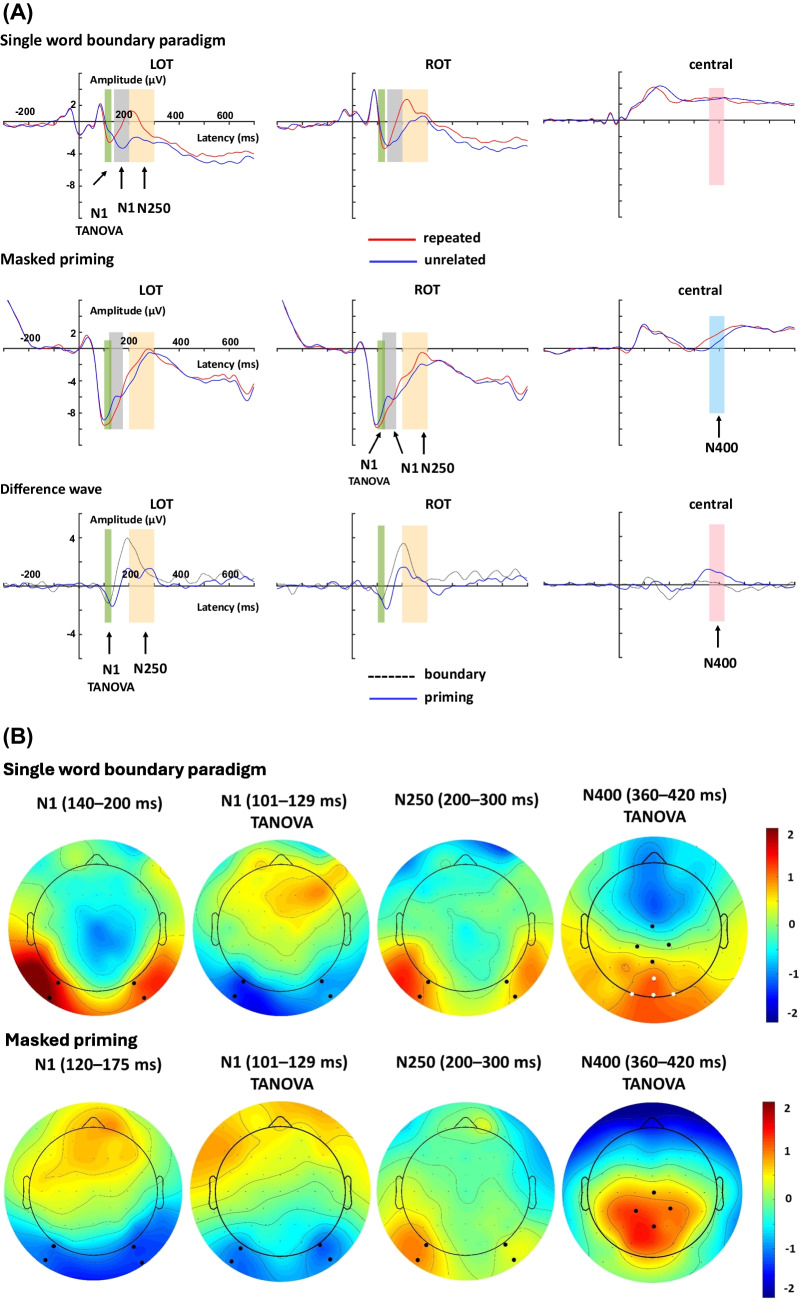
(**A**) Waveforms at left occipital-temporal (LOT), right occipital-temporal (ROT) and central regions for each paradigm. Shading indicates the time windows used for the N1, N250 and N400 components. (**B**) Effect topographies (repeated minus unrelated) of the N1, N250 and N400 repetition effects for each paradigm. Black dots highlight the electrodes used to define the regions of interest (LOT, ROT and central regions), the white dots are the adjusted ROI for the N400 repetition effect for the single word boundary paradigm

Figure 2 presents the electrophysiological results obtained from both the single word boundary paradigm and the masked priming paradigm. To test whether repetition effects can be obtained for each paradigm, we first ran two-way ANOVAs for each time window of interest on the within-subject factors *Repetition* and *Hemisphere*. This was performed for both the N1 and N250 components. Afterwards, three-way ANOVAs on *Paradigm*, *Repetition* and *Hemisphere* were performed to test whether repetition effects differ between paradigms in the two EEG components.

##### N1

In the time-window of the N1 component that we had initially pre-registered for our analysis, we found that both the single word boundary paradigm and masked priming paradigm showed repetition effects (*boundary*: *F*_(1,28)_ = 41.03, *p* < 0.001, ${\eta }_{p}^{2}=0.59$; *masked priming*: *F*_(1,28)_ = 34.41, *p* < 0.001, ${\eta }_{p}^{2}=0.55$, see Fig. 2). However, the repetition effects in the two paradigms were in the opposite directions (increased for repeated characters in the masked priming paradigm and reduced for repeated characters in the single word boundary paradigm compared to unrelated characters). The other main effects and interactions were not significant (*F*s < 0.06, *p*s > 0.81).

We then ran the three-way ANOVAs on *Paradigm*, *Repetition* and *Hemisphere*. As anticipated from the reverse polarity of the effect, results showed that the repetition effects differed between the single word boundary paradigm and the masked priming paradigm (*Paradigm* × *Repetition*, *F*_(1,28)_ = 70.83, *p* < 0.001, ${\eta }_{p}^{2}=0.72$). In addition, masked priming led to a larger, more negative N1 in response to the target than the single word boundary paradigm, regardless of repetition (*Paradigm*, *F*_(1,28)_ = 46.33, *p* < 0.001, ${\eta }_{p}^{2}=0.62$), and the right hemisphere showed larger repetition effects than the left hemisphere (*Repetition* × *Hemisphere*, *F*_(1,28)_ = 12.20, *p* = 0.002, ${\eta }_{p}^{2}=0.304$; left vs. right: −3.83 vs. −4.02 μV). No other interactions were significant (*F*s < 0.005, *p*s > 0.94).

##### N250

In the N250 component, similarly, we ran a two-way ANOVA on *Repetition* and *Hemisphere* for each paradigm separately. Results showed that both the single word boundary paradigm and the masked priming paradigm generated a reduced N250 negativity for repeated characters as compared to unrelated characters (*Repetition*, boundary: *F*_(1,28)_ = 36.62, *p* < 0.001, ${\eta }_{p}^{2}=0.57$; masked priming: *F*_(1,28)_ = 23.36, *p* < 0.001, ${\eta }_{p}^{2}=0.46$). Furthermore, the left hemisphere showed a larger negativity than the right hemisphere, *F*_(1,28)_ = 9.91, *p* = 0.004, ${\eta }_{p}^{2}=0.26$ (left vs. right: −1.23 vs. 0.87 μV). In addition, we observed a tendency that in the single word boundary paradigm, N250 repetition effects were numerically slightly larger (but not significant) in the right as compared to the left hemisphere (*Repetition* × *Hemisphere*, *F*_(1,28)_ = 3.00, *p* = 0.094, ${\eta }_{p}^{2}=0.097$). No other significant main effects and interactions were found (*F*s < 2.05, *p*s > 0.16).

We then ran a three-way ANOVA on *Paradigm*, *Repetition* and *Hemisphere*. The results showed that the masked priming paradigm led to a larger N250 negativity than the single word boundary paradigm (*Paradigm*: *F*_(1,28)_ = 6.58, *p* = 0.016, ${\eta }_{p}^{2}=0.19$), and the N250 was reduced for repeated as compared to unrelated targets (*Repetition*: *F*_(1,28)_ = 41.24, *p* < 0.001, ${\eta }_{p}^{2}=0.60$), Similarly, the left hemisphere showed larger negativity than the right hemisphere (*Hemisphere*: *F*_(1,28)_ = 7.49, *p* = 0.011, ${\eta }_{p}^{2}=0.21$). Interestingly, repetition effects were significantly larger in the single word boundary paradigm than in masked priming (*Paradigm* × *Repetition*: *F*_(1,28)_ = 8.82, *p* = 0.006, ${\eta }_{p}^{2}=0.24$; boundary vs. masked priming: −1.81 vs. −1.01 μV). The left hemisphere showed larger repetition effects than the right hemisphere (*Repetition* × *Hemisphere*: *F*_(1,28)_ = 4.52, *p* = 0.043, ${\eta }_{p}^{2}=0.14$). No other significant main effects and interactions were found (*F*s < 0.22, *p*s > 0.64).

#### TANOVA results

**Fig. 3:**
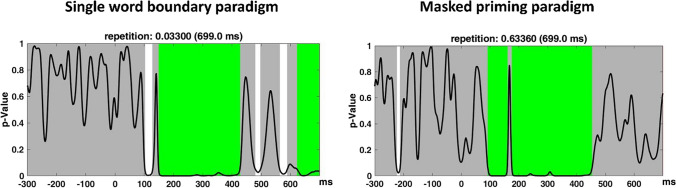
Sample-by-sample TANOVA for the single word boundary paradigm (**left**) and the masked priming paradigm (**right**) with global duration statistics. When using global duration statistics, the duration threshold is used to identify whether an effect is significant. The "duration threshold" denotes the minimum duration that sub-threshold p-values must surpass for an effect to be deemed significant in the overall analysis. This duration is derived from comparing results from various randomization runs. An effect is considered significant when its duration exceeds 95% of all durations obtained under the null hypothesis from random runs. This specific threshold is then utilized in the TANOVA analysis for the corresponding effect. For the masked priming paradigm, the duration threshold was identified to be 51 ms. For the single word boundary paradigm, the duration threshold was identified as 56 ms. The thresholds were then applied to the TANOVA plot, where significant periods longer than this estimated duration threshold are marked in green. The gray areas mark non-significant time points while the white areas mark periods of significant differences between ERP maps of different factor levels

**Fig. 4:**
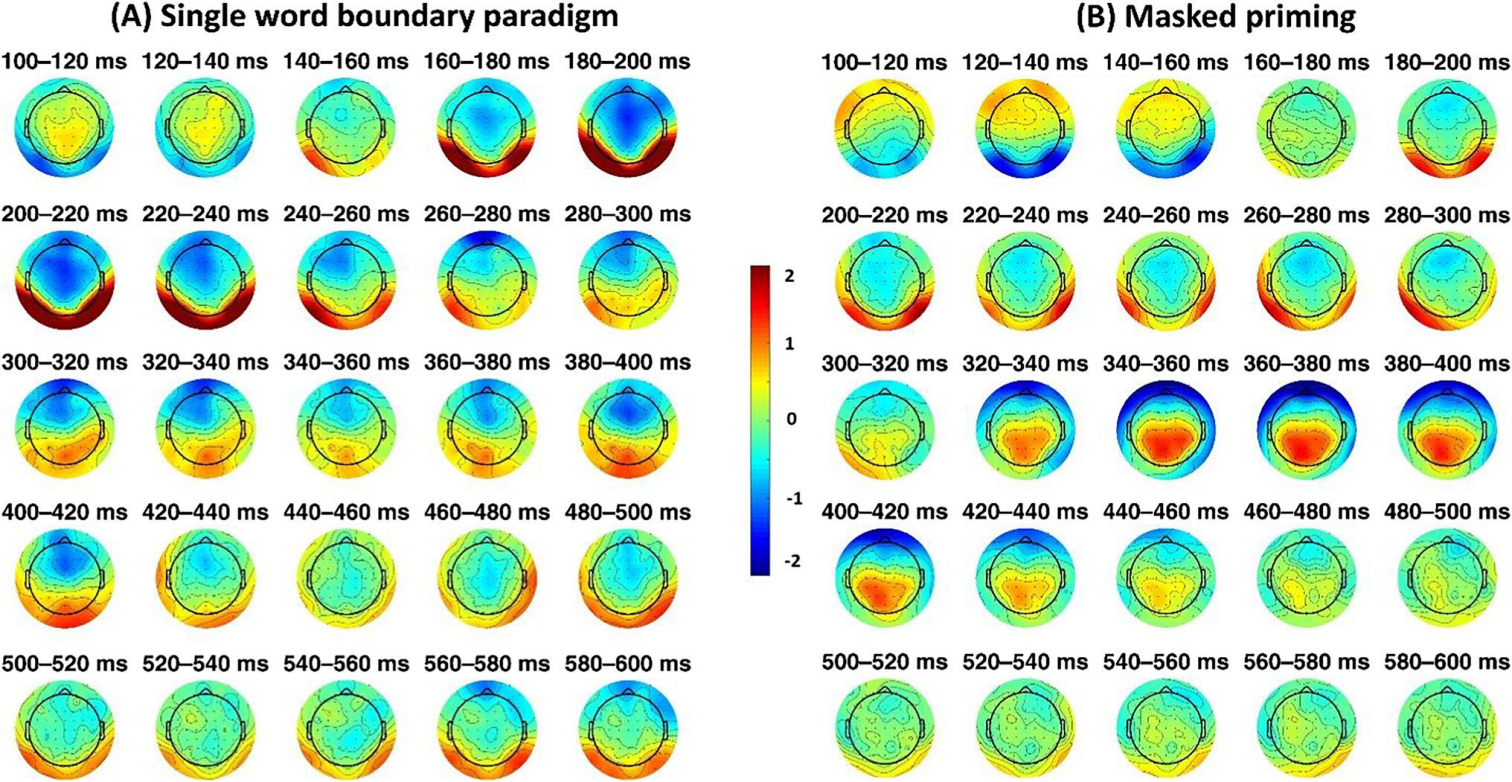
Scalp distribution of effects on the target character (repeated minus unrelated) averaged across consecutive 20-ms windows from 100 to 600 ms. Shown are results for the single word boundary paradigm (**left panel**) and the masked repetition priming paradigm (**right panel**)

Results are shown in Fig. 3. The N1 time window identified in the single word boundary paradigm was 101–129 ms. In the masked priming paradigm it was 93–159 ms. Following the N1, the TANOVA identified a long significant time window in both paradigms, which may reflect an overlap of the N250 and N400 components. This time window was identified from 150 to 427 ms for the single word boundary paradigm, and from 176 to 453 ms for the masked priming paradigm. To further describe the time course and duration of repetition effects in the two paradigms, we plotted additional topographic maps in consecutive 20-ms time windows after fixation/stimulus onset (from 100 to 600 ms; see Fig. 4). Together with the TANOVA results and the topographies, we found that time windows from the preregistration for the N1 overlapped with the N250 effect in both paradigms. Therefore, we ran an additional ANOVA on the N1 component using the time windows identified by TANOVA. We selected the overlapping time windows between paradigms, which were 103–129 ms.

In addition, we also noted that TANOVA revealed robust N400 repetition effects and the repetition effects between the two paradigms showed different topographies, where the N400 repetition effect in the masked priming paradigm has a central-parietal distribution, but this effect in the single word boundary paradigm has a more posterior scalp distribution, so the ROI selected for analysis was based on TANOVA. For the masked priming paradigm, the centroparietal region was selected as the ROI (Cz, CP1, CP2, Pz), whereas for the single word boundary paradigm, the ROI was the posterior region (POz, Oz, O1, O2). Because of the centra scalp distribution for the N400 component, we did not differentiate left and right hemispheres, so the ANOVAs for the N400 component were performed on *Repetition.* Similar to the N1 repetition effect, we used the overlapping time window identified in TANOVA, which is 360–420 ms.

##### N1

The two-way ANOVA on each paradigm showed similar results in the identified N1 time windows (*boundary*: *F*_(1,28)_ = 14.15,* p* = 0.001, ${\eta }_{p}^{2}=0.34$; *masked priming*: *F*_(1,28)_ = 57.76, *p* < 0.001, ${\eta }_{p}^{2}=0.67$), but the direction of the repetition effect in the single word boundary paradigm was reversed to the one in the pre-registered analysis (both paradigms showed increased negativity for repeated targets compared to unrelated targets). No other main effects or interactions were significant (*F*s < 1.38, *p*s > 0.25).

The three-way ANOVA showed significant main effects of *Paradigm* (*F*_(1,28)_ = 48.09, *p* < 0.001, ${\eta }_{p}^{2}=0.63$) and *Repetition* (*F*_(1,28)_ = 11.99, *p* = 0.002, ${\eta }_{p}^{2}=0.30$). However, the interaction between *Paradigm* and *Repetition* was not significant (*F*_(1,28)_ = 64.02, *p* < 0.001, ${\eta }_{p}^{2}=0.70$). In addition, the repetition effect was larger in the left hemisphere than in the right hemisphere (*F*_(1,28)_ = 5.72, *p* = 0.024, ${\eta }_{p}^{2}=0.17$). No other interactions were significant (*F*_(1,28)_ = 2.31, *p* = 0.14, ${\eta }_{p}^{2}=0.08$).

##### N400

In the N400 component, similarly, we examined the repetition effects for each paradigm separately. Results showed that the masked priming paradigm generated a reduced N400 negativity for repeated characters as compared to unrelated characters, but the same result was not found in the single word boundary paradigm (*Repetition*, boundary: *F*_(1,28)_ = 14.36, *p* = 0.001, ${\eta }_{p}^{2}=0.339$; masked priming: *F*_(1,28)_ = 27.18, *p* < 0.001, ${\eta }_{p}^{2}=0.49$ ).

We then ran a two-way ANOVA on *Paradigm* and *Repetition*. The results showed that the masked priming paradigm led to a similar N400 negativity to the single word boundary paradigm (*Paradigm*: *F*_(1,28)_ = 2.25, *p* = 0.15), and the N400 was reduced for repeated compared to unrelated targets (*Repetition*: *F*_(1,28)_ = 42.16, *p* < 0.001, ${\eta }_{p}^{2}=0.60$). However, the repetition effect was not different between paradigms (*Paradigm* × *Repetition*: *F*_(1,28)_ < 0.001, *p* = 0.99; boundary vs. masked priming: 1.07 vs. 1.07 μV).

### Animal detection task and prime/Preview visibility

Participants performed well in the animal task. In the single word boundary paradigm, they detected an average of 96.98% (*d’* = 3.52) of probes in the preview position and 98.56% (*d’* = 4.16) in the target position. In the masked priming paradigm, the mean accuracy of animal probes in the prime position was 96.94% (*d’* = 3.55) and 98.95% (*d’* = 4.33) in the target position.

The d-prime values were calculated from the proportion of hits on trials with animal names in the prime/preview position and false alarms on non-animal prime/preview trials. When the animal probes were in the target position, d-prime was higher than when the probes were in the preview/prime position (*F*_(1,28)_ = 57.09, *p* < 0.001, ${\eta }_{p}^{2}=0.67$). Neither the main effects of *Paradigm* (*F*_(1,28)_ = 0.96, *p* = 0.34) nor the interaction of paradigms and animal positions (*F*_(1,28)_ = 2.35, *p* = 0.13) was significant, suggesting the prime/preview visibility was similar across the two paradigms.

There is evidence suggesting that change awareness affects parafoveal processing (e.g., Angele et al., 2016). This study found that display change detection and lexical processing are not governed by the same cognitive process. White et al. (2005) discovered that subjects with a larger preview benefit effect were more conscious of display changes compared to those who remained unaware. The prime sensitivity may indicate participants' awareness of parafoveal words/primes. Consequently, we correlated this measure to determine if the magnitude of EEG repetition effects among participants was related to their display change sensitivity. Furthermore, to assess any potential associations between the sensitivities in the two paradigms, we also correlated these sensitivities. We found that the d-prime in the preview/prime position was highly correlated with the one in the target position within a paradigm (masked priming: *r* = 0.45, *p* = 0.014; boundary: *r* = 0.63, *p* < 0.001). The N1 preview effects in the single word boundary paradigm were correlated with the d-primes in the preview and target position, but the other EEG repetition effects were not correlated with the d-primes (see Table 1). However, it should be noted that all these significant correlations were not significant after Bonferroni corrections except the one for the d-prime in the preview position and target position of the single word boundary paradigm.

**Table 1:** Correlations between repetition effects in the N1 and N250 components and the d-prime when animal characters were in the prime/preview position or target position

	B: N1	MP: N1	B: N250	MP: N250	*d’*: B-preview	*d’*: B-target	*d’*: MP-prime	*d’*: MP-target
B: N1	1	0.259	0.265	0.32	**−0.49**	**−0.384**	0.071	0.059
MP: N1		1	-0.228	−0.041	−0.194	−0.299	−0.248	−0.114
B: N250			1	**−0.478**	−0.234	0.004	0.049	0.151
MP: N250				1	−0.126	−0.31	0.040	−0.191
*d’*: B-preview					1	0.632^**^	0.306	0.035
*d’*: B-target						1	0.085	0.345
*d’*: MP-prime							1	**0.453**
*d’*: MP-prime								1

^**^ Correlation is significant at the 0.001, 0.0006, and 0.001 level (2-tailed) after Bonferroni corrections, respectively, ordered from left to right and from top to bottom

*Note.* B represents the single word boundary paradigm, and MP represents masked repetition priming paradigm; we used different alpha levels for Bonferroni correction for the three rectangle regions as we actually examined three different correlation relationships. The upper left rectangle region represents the correlations among the N1 and N250 repetition effects for the single word boundary and masked priming paradigm. The upper right rectangle region represents the correlations between the EEG repetition effects and the d-primes. The lower right rectangle region represents the correlations among the d-primes of masked priming and single word boundary paradigm. Specifically, the correlation coefficients in bold indicate that significant correlations were observed prior to applying the Bonferroni corrections, but not after

### Display change awareness

Our measure of display change awareness assessed whether participants noticed that the display was manipulated. As expected, the display change awareness results showed that almost all participants were aware of the change of preview/prime, except three participants who reported they did not note any change in the masked priming experiment. They reported a higher total number of trials in the single word boundary paradigm than in the masked priming paradigm (boundary: *M* = 52.28, *SD* = 27.44; masked priming: *M* = 49.42, *SD* = 30.84). For the number of trials with perceived changes, the participants reported an average of 29 (boundary: *M* = 29.38, *SD* = 21.8; masked priming: *M* = 29.48, *SD* = 23.64) trials that showed changes (actually there were 200 changes in each paradigm, including 100 trials that involved only a font change but the same word). Therefore, participants reported more frequent changes that occurred in the masked priming paradigm than in the single word boundary paradigm, as participants reported a larger percentage of changed trials in the masked priming paradigm than in the single word boundary paradigm (boundary vs. masked priming: 58.04% vs. 64.48%). This is likely explained by the inclusion of both a forwards and a backward mask making the screen changes more frequent.

## Discussion

The current study directly compared the neural correlates underlying the effects of masked priming and parafoveal preview by presenting the same Chinese single character to the same participants in both paradigms. In each case, previews/primes were either identical or unrelated to the target characters. The neural correlates were measured by means of co-registering EEG and eye movements. We discuss the main findings below, beginning with the N250, followed by the N1 and N400 components.

### N250 repetition effect

In the time window from 200–300 ms, we observed repetition effects in both the single word boundary paradigm and the masked priming paradigm. Although the effect was larger in the single word boundary paradigm, the scalp distribution (occipital-temporal regions) and time course (200–300 ms) were similar in both paradigms. The preview positivity in the boundary paradigm has been suggested to reflect the preview-based facilitation of early stages in visual word recognition at visual feature and/or orthographic levels (Niefind & Dimigen, 2016). Consistent with this hypothesis, most eye-movement researchers have located preview benefits in fixation durations primarily at the orthographic level (and to a lesser degree at the phonological level) (Schotter et al., 2012b; Vasilev & Angele, 2017b). Also, in some EEG studies on orthographic processing, a separable N250 component was identified in addition to the N1(Dufau et al., 2008; Hauk & Pulvermüller, 2004; Holcomb & Grainger, 2006; Kiyonaga et al., 2007), whereas in other studies, the N1 showed a decreasing flank that also covered the N250 time range (Dimigen et al., 2012; Eberhard‐Moscicka et al., 2015; Huang et al., 2022; Li et al., 2015; Wang & Maurer, 2020). In addition, the time point-wise TANOVA revealed that the preview positivity starts around 150 ms after fixation onset. This result is consistent with the idea that saccade preparation in reading was estimated to take about 150–175 ms (Rayner et al., 1983). Thus, the temporal finding of the preview positivity is a plausible correlate of the facilitating brain processes that lead to the preview benefit in behaviour.

The N250 repetition effects in the masked priming paradigm has been suggested to reflect a process at the interface between sub-lexical and whole word representations and is sensitive to the degree of prime-target orthographic overlap; both partial repetitions and pseudoword repetitions benefit from the orthographic overlap between prime and target (for a review, see Grainger & Holcomb, 2010). The repetition effects in the boundary paradigm have been suggested to reflect a form of partial, trans-saccadic repetition priming that activates abstract orthographic and phonological representations (Dimigen et al., 2012). Given previous interpretations of these effects, it is possible that the preview positivity and the N250 repetition effects reflect a similar process in visual word recognition in the time window of 200–300 ms.

However, as mentioned above, we found evidence that the N250 repetition effect is larger in the single word boundary paradigm than in masked priming. This phenomenon is consistent with the findings of Kornrumpf et al. (2016), who compared ERPs in an RSVP-with-flanker paradigm (i.e., RSVP presentation with parafoveal flanker words providing previews) and FRPs in the boundary paradigm. They found that the preview positivity (200–280 ms) was substantially smaller in ERPs (without eye movements) than in FRPs. This may be due to the mandatory shift of visuo-spatial attention toward *n* + 1 occurring in natural reading that is coupled to the preparation of the saccade. In contrast, such an attention shift is likely unnecessary in the RSVP-flanker paradigms in which participants maintain constant eye fixation while words are presented one by one in foveal vision at a fixed pace (e.g., masked priming paradigm). Therefore, we think that the basic processes reflected by the N250 masked priming effect and the preview positivity may be very similar, but that they are more pronounced in the latter paradigm, possibly because of the deep level of consciousness of the stimuli, specific word parts that readers process (e.g., word centre vs. initial letters), the active/passive eye movement between paradigms that could further influence the timing of cognitive processes or their modulation by attention, or the duration of the previews/primes. It has been shown that prime duration affects repetition effects, both in brain activity (Holcomb et al., 2005) and in performance (Angele et al., 2022). Thus, longer preview durations enhance preview effects (Yan et al., 2012a, 2012b). In our study, while prime duration was 50 ms, preview durations often exceeded 150 ms. It is conceivable that this disparity in prime/preview duration might lead to the earlier N1 effect in the single word boundary paradigm compared to the masked priming paradigm, since longer previews may accelerate early visual processing.

Although many researchers agree that the N250 effects between 200 and 280 ms reflects preview benefits, it is under debate that the N250 effects may be also the consequence of preview costs, as readers may generate implicit expectations about the upcoming words. How to explain the effect is actually determined by the baseline used for computing the preview effects (e.g., Yan et al., 2012a, 2012b). However, this argument can be viewed as two sides of the same coin within the predictive coding framework (e.g., Summerfield et al., 2008). The framework of predictive coding (Friston, 2005) assumes that humans constantly generate predictions about likely sensory inputs, whereas the actual sensory input serves as an error signal to allocate attention to unpredicted events. Within this framework, expectations before the saccade may be violated after the saccade following invalid previews (as suggested by Kornrumpf et al., 2016). Furthermore, some studies have found that the N250 modulations showed some resemblance to the visual MMN (Stefanics et al., 2014), thereby reflecting a mismatch signal (Rao & Ballard, 1999). When the upcoming words fulfil the expectations (i.e., identical previews/primes), the processing is facilitated.

### N1 repetition effect

In the masked priming paradigm, we obtained a typical repetition effect in the N1 component (e.g., Petit et al., 2006), where targets elicited larger amplitudes after identical than after unrelated primes at occipital-temporal electrodes. While the timing of this effect coincides with a broad negativity in the ERP with several small peaks, the early time window of this effect around 100–150 ms may correspond to the P1-N1 transition in regular ERPs, where the P1 typically occurs around 100 ms, followed by the N1 at around 150 ms (e.g., Grainger & Holcomb, 2009). The repetition priming effect in this time window therefore would also be in agreement with a forward-shift of the N1 component latency, although this might not be visible in masked priming experiments due to the overlap with ERP components elicited by the preceding mask and prime. This speculation might be examined by deconvolution to resolve the overlap of brain responses to different event types (e.g., Ehinger & Dimigen, 2019; Smith & Kutas, 2015a, 2015b).

The TANOVA results revealed that the N1 repetition effects in the single word boundary paradigm occurred earlier (i.e., 103–129 ms) than the time window proposed in our pre-registration (140–200 ms). This earlier time window identified by the TANOVA may be due to different approaches for identifying time windows. In the pre-registration, we selected the time window of the N1 repetition effect based on previous studies (Dimigen et al., 2012), but the data-driven approach can capture the effect more precisely.

Previous studies, such as by Niefind and Dimigen (2016), have also reported relatively early N1 preview effects, finding an early identity preview effect with a larger negativity after unrelated rather than valid previews. However, this direction of effect was more consistent with the typical preview positivity in the later time window (200–300 ms). Therefore, the previous reports of N1 preview effects between 160 and 200 ms should be considered as an early part of preview positivity, rather than early N1 effects. The TANOVA results support the hypothesis, showing that a positive preview effect began as soon as 153 ms after the start of fixation. These findings provide further insights into the mechanisms underlying the classic preview effect (Degno et al., 2012). In contrast, our early N1-like preview effect has a similar scalp distribution and the same polarity of effect as the N1 repetition effect in the masked priming paradigm, and may reflect the feature overlap processing between prime and target. When targets and previews/primes are identical, the targets share a larger number of visual features with the primes or previews as compared to mismatched pairs, and targets after identical primes/previews have a processing benefit. Therefore, we believe that the N1 effects observed in the masked priming and single word boundary paradigms reflect the same mechanism of perceptual priming at the visual feature level.

The early N1 effect might also be influenced by visual crowding and masking. Degno et al. (2019a) demonstrated this in a study using the boundary paradigm, where they modified the target's preview and the inter-word spacing. When a random letter replaced the inter-word space (crowding), the onset of the early N1 preview effect was delayed relative to conditions with an intact inter-word space. In contrast, the N250 component was less influenced by visual crowding.

In addition, we think that the pattern of an N1 increase and a N250 decrease (e.g., also qualitatively seen in Dimigen & Ehinger, 2021; see their Fig. 8) could potentially occur due to a latency shift of the N1 after valid previews or primes. However, in this case, the size of the N1 effect should correspond to the size of the N250 effect, which was not the case in the current study. So, a potential latency shift could only partially explain the N250 effect. Therefore, one speculation is that the facilitation effect does not just seem to be the result of speeding up N1 processing, but presumably it also consists of reduced activation during the N250 time range or potentially acceleration of subsequent processing.

### N400 repetition effect

For the N400 component, the repetition effects were similar across the two paradigms, but they showed different topographies. Specifically, the N400 effect in the masked priming paradigm was more central-parietal distributed, whereas the effect in the single word boundary paradigm was more posterior distributed. These slight differences in scalp topography may reflect the conscious perceptibility or/and the selective attention differences across the two paradigms. A previous study has found that the central-parietal N400 effect is associated with automatic lexical priming, while the posterior N400 effect may reflect semantic voluntary, effortful integration process (Schöne et al., 2018). In the masked priming paradigm, the primes are less visible as compared to the previews in the single word boundary paradigm, and the differences of spatial location of the prime/preview may also influence the attention allocation, which further influence semantic retrieval (Van Berkum, 2009). In addition, the TANOVA analysis revealed that the latency of the N400 effect in the masked priming paradigm is longer than in the boundary paradigm, which may be due to the larger N250 effect in the single word boundary paradigm, resulting in the more efficient process of lexical processing.

### Effects of prime/Preview visibility on ERP measures

In the masked priming paradigm, neither the early N1 nor the N250 repetition effect were influenced by the prime’s visibility – i.e., whether the participant could correctly classify whether this briefly presented and masked word was an animal or not; in contrast, in the single word boundary paradigm, preview visibility seems to influence the N1 preview effect, but not the later N250 effect. A previous masked priming study (Holcomb et al., 2005) found that prime visibility in a masked priming paradigm only influenced the N400 but not earlier effects (e.g., N250), suggesting that prime visibility may have little influence on early stages of visual word recognition for the target word. In a masked priming paradigm, where readers fixate on the centre of the screen, the primes are effectively masked to the greatest degree. In contrast, in a boundary paradigm, readers are allowed to move their eyes freely. In this case, when the participants’ task is to judge whether there was an animal probe in a trial, they may develop a strategy to move their eyes to the target word later and use their extrafoveal vision to try to identify the preview. This hypothesis is supported by the more robust correlation between the N1 preview effect and sensitivity (d-prime) when the animal probe was in the preview position than the one when the animal probe was in the target position. This may explain why the preview visibility only influences the repetition effects in the single word boundary paradigm but not in the masked repetition priming paradigm. In addition, the N1 component is sensitive to the degree of overlap between the target and prime items, while the N250 component is more sensitive to orthographic information. The prime visibility might depend more on the visual overlap between the preview and target than on their orthographic overlap. Moreover, Angele et al. (2016) reported that awareness of display changes may emerge during the early pre-attentional stages. Lexical processing, which occurs later, may not require attention. Therefore, display change awareness could affect the early stage (i.e., N1) rather than the later stage (i.e., N250). This may explain why preview visibility influenced only the early N1 preview effect, not the later component.

Another interesting finding from the correlation analyses was that, across participants, the size of the N250 preview effect in the single word boundary paradigm correlated with the size of the N250 repetition effect in the masked priming paradigm. The results provide further support that these two effects have a similar neural basis; this holds true even though the N250 effect was larger in the single word boundary paradigm than in the masked priming paradigm.

### Limitations and future directions

While our approach offers a unique perspective on the intrinsic aspects of parafoveal processing, we are aware of some limitations of the current study. First, we focused on the neural mechanisms involved in recognizing individual words, as opposed to reading typical text. This approach does not only omit contextual and semantic aspects, which are known to influence not only word recognition itself (refer to Staub, 2015, for eye-movement behaviour implications, and Kutas & Federmeier, 2011, and Federmeier, 2022, for ERP impacts), but also the dynamics of parafoveal preview effects (e.g., Andrews & Veldre, 2019; Schotter, 2018). Additionally, the preview effects observed in standard reading paradigms do not exclusively represent repetition (also known as trans-saccadic integration). Thus, preview validity is not the sole factor of importance. Consequently, any attempt to apply these findings to conventional reading practices should be undertaken with caution.

A potential issue arises with the high display change awareness in our study, which might influence parafoveal processing. Angele et al. (2016) discerned that readers exhibit different sensitivities when previews are nonword-like as opposed to word-like. White et al. (2005) discovered that subjects who showed greater preview benefit effects were more aware of display changes than those who were less aware. In contrast, Dimigen (2012) showed that robust N1/N250 preview effects in FRPs are observed both in readers who are unaware of display changes, and in readers who noticed some proportion of the changes during the experiment. In our study, the awareness of display changes across both paradigms was notably pronounced, perhaps due to the shift from parafoveal stimuli/primes to targets and the stronger visual changes in the masked priming paradigm (e.g., the masks presented). To address this matter, subsequent studies might ask participants to affirm if they detected a display change right after each trial.

Third, we noted that the number of remaining trials in the single word boundary paradigm was smaller than that in the masked priming paradigm, which means that our analysis might be less sensitive to pick up effects in the single word boundary paradigm. One reason is that there were generally more EMG artefacts in the boundary paradigm. Another reason for the larger loss of trials was premature saccades in some trials, which occurred before the preview was even presented. These early eye movements might have resulted from the rather long fixation check at the beginning of each trial. However, even though fewer trials were left for analysis of the single word boundary paradigm compared to the masked priming paradigm, we still observed a larger N250 repetition effect in the boundary paradigm, suggesting that the effect is robust^2^

Fourth, as mentioned earlier, we did not find a preview effect in fixation times in our less natural reading setting. We chose this single-saccade version of the boundary paradigm in order to make the two paradigms directly comparable. Future studies may consider an experimental design closer to a natural reading situation, for example, by embedding the words into sentences or word lists, or by adding another fixation point to the right of the targets, so there is also an outgoing saccade from the target word.

In a masked repetition priming paradigm, the prime is backward-masked after a brief foveal presentation, which renders it invisible. In contrast, in the single word boundary paradigm, the preview is presented parafoveally but usually with a longer duration (corresponding to the gaze duration on the fixation cross). Although the N250 effects differed in size, the current results suggest that the consequences of these two rather different manipulations are qualitatively surprisingly similar at the neural level. Future studies could address in more detail the question of to what degree a brief backward-masked prime is comparable to a longer but parafoveally degraded and visually crowded preview and at which level the previews or primes are processed. This could be done by introducing new experimental conditions, for example, by shifting the prime into parafoveal vision in the masked priming paradigm (Pernet et al., 2007) or by masking the preview in the boundary paradigm (cf., Hohenstein et al., 2010; Rayner et al., 2006).

## Conclusion

In sum, qualitatively similar effects on the N1 and N250 components were obtained in the single word boundary paradigm and the masked priming paradigms. The similar scalp distributions, time courses, and the directions of effects indicate that the neural mechanisms underlying the two paradigms are similar or strongly overlapping. Furthermore, the use of the single word boundary paradigm, which allows readers to execute saccades, had a bigger impact on the N250 component as compared to the very early stage of visual word recognition, possibly because readers are more actively engaged in reading.

## Supplementary Information

Below is the link to the electronic supplementary material.

Supplementary file1 (DOCX 1816 KB)

## Data Availability

All data and codes have been made publicly available via the Open Science Framework at https://osf.io/hk37q/.

## Footnotes

1 Note that we accidently set the duration of the target presentation to 1,000 ms and therefore longer than the target duration in the masked priming paradigm. This is likely not crucial, as we exclusively analyze effects that occur well before 500 ms. However, to further test whether the duration of target had any substantive influence on the early electrophysiological effects, we ran a supplementary study in which the duration of targets was set to 500 ms in both paradigms. This is reported as a supplementary experiment in the OSM.

2 We conducted an additional analysis where we randomly removed some trials from the masked priming paradigm to equalize the number of trials analyzed between the two paradigms. The outcome patterns remained consistent. For more details, please refer to the OSM.
