# Supplementary material for: Neural mechanism underlying preview effects and masked priming effects in visual word processing
Source: Atten Percept Psychophys. 2024 Jul 2;87(1):5–24. doi: 10.3758/s13414-024-02904-8 (PMC11845427; doi:10.3758/s13414-024-02904-8)
Supplement: Supplementary file 1 — Supplementary file1 (DOCX 1816 KB) [file 13414_2024_2904_MOESM1_ESM.docx]

# Supplementary experiment

During the main experiment, the targets in the single word boundary paradigm were accidently presented for 1000 ms rather than 500 ms (as in the masked priming paradigm). Because all ERP components we focused occurred before 500 ms, we think this would not influence the results much. Nevertheless, to make the two paradigms even more comparable, we ran an additional experiment in which we set the duration of the target presentation in both paradigms to 500 ms.

## Participants

An additional 16 native Cantonese Chinese participants (7 females; mean age = 20.55 years, range = 18–22 years) were tested in both experiments. Recruitment criteria for participants were the same as in the proper experiment. Data from three subjects were excluded from the analysis due to a small number of remaining trials (*n* < 15).

## Materials and procedure

All materials and procedures were identical to the main experiment, except that the duration of the target presentation in the single word boundary paradigm (relative to the eyes triggering the invisible boundary) was set to 500 ms.

## Results

### ***Traditional ANOVA results***

We took the same strategy in the formal experiment to analyze the data from this supplementary experiment. We first run two-way ANOVAs for each time window of interest with within-subject factors *Repetition* (unrelated vs. repeated), and *Hemisphere* (left vs. right).

**N1.** For the N1 component, we found that both the single word boundary paradigm and masked priming paradigm found repetition effects (*boundary*: *F* _(1,12)_ = 12.90, *p* = 0.001, 0.52; *masked* *priming*: *F* _(1,12)_ = 20.99, *p* = 0.001, 0.64). Similar to the formal experiment, we observed that the repetition effects between the two paradigms were opposite (increased for repeated characters in the masked priming paradigm and reduced for repeated characters in the single word boundary paradigm compared to unrelated characters). In addition, the repetition effect was slightly larger in the right hemisphere compared to the left hemisphere (*Repetition* × *Hemisphere*, *F* _(1,12)_ = 3.84, *p* = 0.074, 0.24). The other main effects and interactions were not significant (*F*s < 1.51, *p* > 0.24).


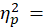

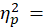

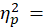

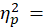

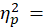

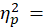


We then ran the three-way ANOVAs on *Paradigm*, *Repetition* and *Hemisphere*. Results showed that repetition effects were larger in the single word boundary paradigm than that in the masked priming paradigm (*Paradigm* × *Repetition*, *F* _(1,12)_ = 30.75, *p* < 0.001, 0.72). In addition, the masked priming led to larger N1 negativity in the subsequent targets than the single word boundary paradigm (*Paradigm*, *F* _(1,12)_ = 34.94, *p* < 0.001, 0.74), and the right hemisphere showed slightly larger activation for repetition effects than the left hemisphere (*Repetition* × *Hemisphere*, *F* _(1,12)_ = 3.28, *p* = 0.095, 0.215; left vs. right: –3.32 vs. –3.82 µV). No other interactions were significant (*F*s < 2.16, *p* > 0.17).


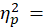

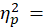

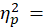

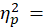

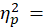

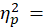


**N250.** For the N250 component, similarly, we ran a two-way ANOVA on *Repetition* and *Hemisphere* for each paradigm. Both the single word boundary and masked priming paradigms revealed reduced N250 negativity for repeated characters compared to unrelated characters (*Repetition*, boundary: *F* _(1,12)_ = 4.17, *p* = 0.064, 0.26; masked priming: *F* _(1,12)_ = 3.23, *p* = 0.097, 0.21). In addition, we observed that in the masked priming paradigm, the repetition effects were slightly larger in the right hemisphere than left hemisphere with a marginally significant interaction between *Repetition* and *Hemisphere* (*F* _(1,12)_ = 3.30, *p* = 0.095,
0.22). No other significant main effects and interactions were found (*F*s < 1.68, *p* > 0.22).


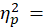

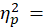

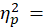

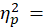


We then ran the three-way ANOVA on *Paradigm*, *Repetition* and *Hemisphere*. A larger N250 negativity in the subsequent targets was found in the masked priming paradigm than the single word boundary paradigm (*Paradigm*, *F* _(1,12)_ = 18.80, *p* = .001, 0.61), and the N250 was reduced for repeated compared to unrelated targets (*Repetition*, *F* _(1,12)_ = 4.96, *p* = 0.048, 0.29). The left hemisphere showed larger activation for repetition effects than the right hemisphere, *Repetition* × *Hemisphere*, *F* _(1,12)_ = 3.95, *p* = 0.07, 0.25. No other significant main effects and interactions were found (*F*s < 0.39, *p*s > 0.55).


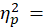

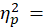

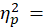

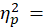

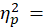

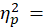


### ***TANOVA results***

Similar to the formal experiment, we ran a time-point-wise TANOVA comparing repeated and unrelated targets for each paradigm. For the masked priming paradigm, TANOVA identified 4 time windows after stimulus onset, which are 113–152 ms (N1), 194–215 ms (N250), 316–457 ms and 606–642 ms. For the single word boundary paradigm, similarly, 4 time windows were identified, which are 103–129 ms (N1), 162–217 ms (N250), 325–349 ms, and 369–386 ms (see Supplementary Figure 1A).

As TANOVA results revealed, the N250 repetition effects were narrowed in a shorter time window for both paradigms and no longer overlapped with the time window of the N400 effect. Therefore, we further tested the N1, N250 and N400 effects with the time windows identified by TANOVA, with overlapping time windows between paradigms. Therefore, for the N1 component, the time window for analysis was 103–129 ms; for N250 component, the time window was 194–215 ms; for N400 component, the time window was 325–386 ms.

**N1.** Similar to the traditional analyses, we ran the two-way ANOVA on Repetition and Laterality for each paradigm. Results indicated that the single word boundary and masked priming paradigms both showed increased N1 negativity for repeated characters, in contrast to unrelated characters (*Repetition*, *boundary*: *F* _(1,12)_ = 14.78, *p* = 0.002, 0.55; *masked priming*: *F* _(1,12)_ = 15.71, *p* = 0.002, 0.57). The right hemisphere showed larger activation than the left hemisphere for the repetition effects in the single word boundary paradigm *(Hemisphere*, *F* _(1,12)_ = 7.17, *p* = 0.02, $\mathrm{QUOTE}$ 0.37). In addition, for both paradigms, the repetition effects were more robust in the left hemisphere than right hemisphere (*masked priming*: *F* _(1,12)_ = 5.70, *p* = 0.034, $\mathrm{QUOTE}$ 0.32; *boundary*: *F* _(1,12)_ = 5.07, *p* = 0.044, 0.30).


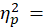

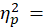

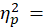

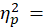

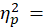

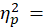


The three-way ANOVA on *Paradigm*, *Repetition* and *Hemisphere* revealed no significant differences in the repetition effects between the two paradigms (*Repetition* × *Paradigm*, *F* _(1,12)_ = 0.24, *p* = 0.64, 0.02). In addition, larger N1 negativity was found in the masked priming than single word boundary paradigm (*F* _(1,12)_ = 25.15, *p* < 0.001, 0.68). Repeated targets elicited larger negativity than unrelated targets (*F* _(1,12)_ = 39.40, *p* < 0.001, 0.77). In addition, the single word boundary paradigm showed larger hemisphere differences (*Paradigm* × *Hemisphere*, *F* _(1,12)_ = 5.33, *p* = 0.04, 0.31). Most importantly, we observed a three-way interaction between *Repetition*, *Paradigm* and *Hemisphere* (*Paradigm* × *Hemisphere* × *Repetition*, *F* _(1,12)_ = 17.94, *p* < 0.001, 0.60). This interaction indicated that the repetition effect was more pronounced in the right hemisphere for the masked priming paradigm, while in the single word boundary paradigm, it was larger in the left hemisphere. The other main effects or interactions were not significant (*F*s < 1.07, *p*s > 0.32).


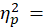

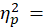

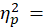

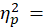

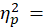

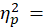

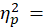

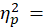

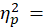

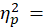


**N250.** The two-way ANOVA on *Repetition* and *Hemisphere* for each paradigm showed that a reduced N250 negativity for repeated characters compared to unrelated characters was found in both the single word boundary paradigm and masked priming paradigm (*Repetition*, boundary: *F* _(1,12)_ = 22.36, *p* < 0.001, 0.65; masked priming: *F* _(1,12)_ = 11.25, *p* = 0.006, 0.48). The other main effects or interactions were not significant (*F*s < 2.59, *p*s > 0.14).


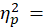

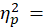

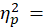

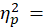


The three-way ANOVA on *Paradigm*, *Repetition* and *Hemisphere* did not reveal any interactions, although the repetition effects in the single word boundary paradigm were larger compared to the masked priming paradigm (*Paradigm* × *Repetition*, *F* _(1,12)_ = 0.83, *p* = 0.38, 0.06, mean amplitude: 2.14 vs 1.63). The repetition effect was significant (*F* _(1,12)_ = 25.18, *p* < 0.001, 0.68), with repeated targets eliciting reduced negativity compared to targets after unrelated primes/previews. Also, the amplitudes in the masked priming paradigm were larger than that in the single word boundary paradigm (*Paradigm*, *F* _(1,12)_ = 20.44, *p* = 0.001, 0.63).


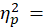

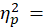

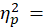

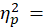

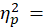

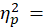


**N400.** The TANOVA results revealed a difference in the scalp distribution of the repetition effect. Although the ROI used in our proposed analysis for masked priming aligned with the TANOVA analysis, the TANOVA pinpointed the frontal-central region (FCz, Cz, C1, C2) as showing the largest significant activation. Consequently, using the time windows identified for the N400 from the TANOVA, we performed the ANOVA analysis. In the N400 component, we examined the repetition effects for each paradigm separately. Results showed significant repetition effects for both paradigms. For the single word boundary paradigm, there was a reduced N400 negativity for repeated characters compared to unrelated characters (*F* _(1,12)_ = 12.30, *p* = 0.004, 0.51). Similarly, in the masked priming paradigm, repeated characters showed reduced N400 negativity compared to unrelated characters (*F* _(1,12)_ = 25.58, *p* < 0.001, 0.70).


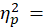

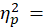

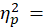

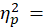


We conducted a two-way ANOVA with factors *Paradigm* and *Repetition*. The results revealed that the N400 negativity was marginally larger in the single word boundary paradigm compared to the masked priming paradigm (*Paradigm*: *F* _(1,12)_ = 8.04, *p* = 0.015, 0.40). Additionally, the N400 was reduced for repeated targets compared to unrelated targets (*Repetition*: *F* _(1,12)_ = 47.30, *p* < 0.001, 0.80). While the repetition effect in the masked priming paradigm was larger than in the single word boundary paradigm, this difference was not statistically significant (*Paradigm* × *Repetition*: *F* _(1,12)_ = 3.24, *p* = 0.097, 0.21; boundary vs. masked priming: 0.72 vs. 1.36 μV). The repetition effects between the two paradigms were similar in size but exhibited slight differences in the scalp distribution. In light of the findings from the main experiment, the N400 repetition effect in the single word boundary paradigm was more sensitive to individual differences. However, this effect in the masked priming paradigm remained uninfluenced by the sample.


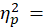

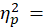

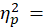

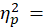

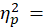

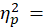


In summary, a small group of 13 participants showed consistent repetition effects across both paradigms for all three components. For the N1 and N400 components, the differences in repetition effects aligned with the findings of the main experiment. However, for the N250 component, we did not find a larger repetition effect in the single word boundary paradigm compared to the masked priming paradigm. The nonsignificant interaction might be attributed to the limited sample size. Importantly, the repetition effects remained largely unaffected by variations in target duration.


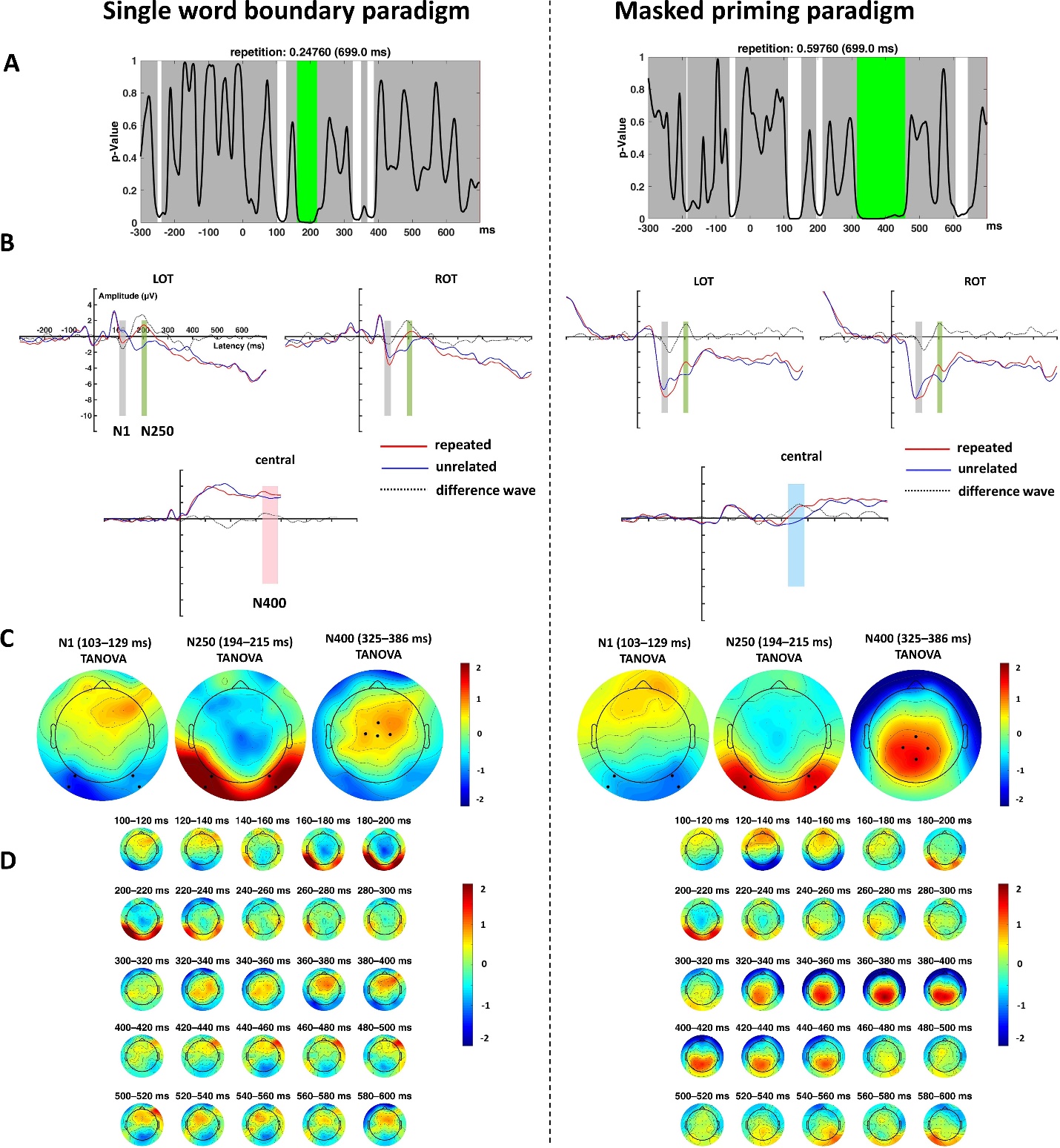


*Supplementary Figure 1.* Detailed results for the preview effects (left panel) and masked priming repetition effects (right panel). (A) TANOVA results with global duration statistics correction. For the single word boundary paradigm, the duration threshold was identified as 45 ms. For the masked priming paradigm, the duration threshold was identified as 45 ms. The thresholds were then applied to the TANOVA plot, where periods longer than the estimated duration threshold are marked in green. (B) Repetition effects (repeated minus unrelated) in left occipital–temporal (LOT, PO7 and PO9) regions, right occipital–temporal (ROT, PO8 and PO10) and central-parietal (Cz, CP1, CP2, Pz) regions for the single word boundary paradigm (left panel) and masked priming paradigm (right panel). (C) Topography maps of repetition effect (repeated minus unrelated) in N1, N250 and N400 for each paradigm. The black dots indicate the regions of interest (ROIs) in the main analysis, while the white dots indicate the time window after TANOVA adjustment. (D) Temporal evolution of repetition effect (repeated minus unrelated) in successive 20 ms time windows between 0 and 600 ms after stimulus/fixation onset.

Supplementary Table 1

Table 1. *Table of ANOVA results on the ERP/FRP data with equal trials between the two paradigms*

| Component | Factor | *F* | *p* |
| --- | --- | --- | --- |
|  | Repetition | 36.14 | <0.001*** |
|  | Paradigm | 72.26 | <0.001*** |
| N1 | Hemisphere | 0.01 | 0.93 |
|  | Repetition × Paradigm | 0.20 | 0.66 |
|  | Repetition × Hemisphere | 0.96 | 0.34 |
|  | Paradigm × Hemisphere | 0.15 | 0.70 |
|  | Repetition × Repetition × Hemisphere | 0.69 | 0.41 |
| N250 | Repetition | 36.86 | <0.001*** |
|  | Paradigm | 7.13 | 0.012* |
|  | Hemisphere | 7.52 | 0.01* |
|  | Repetition × Paradigm | 5.48 | 0.026* |
|  | Repetition × Hemisphere | 2.49 | 0.13 |
|  | Paradigm ×Hemisphere | 3.66 | 0.66 |
|  | Repetition × Repetition × Hemisphere | 0.77 | 0.38 |
| N400  (TANOVA adjusted) | Repetition | 5.49 | 0.03 |
|  | Paradigm | 2.36 | 0.14 |
|  | Repetition × Paradigm | 0.08 | 0.78 |

+ *p* < 0.1. * *p* < 0.05. ** *p* < 0.01. *** *p* < 0.001.

Supplementary Table 2

Table 2. *Fixed Effect Estimates from the Linear Mixed-Effects Models on the EEG Data*

| Component | Factor | *b* | *CI* | *p* |
| --- | --- | --- | --- | --- |
| N1 | (Intercept) | -0.90 | −2.23 – 0.43 | 0.184 |
|  | Repetition | −1.09 | −1.63 – −0.55 | <0.001*** |
|  | Paradigm | −5.49 | −5.95 – −5.02 | <0.001*** |
|  | Hemisphere | −0.04 | −0.54 – 0.47 | 0.888 |
|  | Repetition × Paradigm | 0.18 | −0.48 – 0.83 | 0.598 |
|  | Repetition × Hemisphere | 0.15 | −0.56 – 0.86 | 0.686 |
|  | Paradigm ×Hemisphere | 0.03 | −0.62 – 0.69 | 0.917 |
|  | Repetition × Repetition × Hemisphere | −0.40 | −1.33 – 0.53 | 0.398 |
| N250 | (Intercept) | −2.23 | −3.81 – −0.66 | **0.005**** |
|  | Repetition | 2.03 | 1.48 – 2.58 | **<0.001***** |
|  | Paradigm | −0.86 | −1.35 – −0.37 | **0.001***** |
|  | Hemisphere | 2.23 | 1.75 – 2.72 | **<0.001***** |
|  | Repetition × Paradigm | −0.91 | −1.60 – −0.22 | **0.009**** |
|  | Repetition × Hemisphere | −0.40 | −1.08 – 0.29 | 0.254 |
|  | Paradigm ×Hemisphere | −1.25 | −1.94 – −0.56 | **<0.001***** |
|  | Repetition × Repetition × Hemisphere | 0.26 | −0.71 – 1.23 | 0.599 |
| N400  (TANOVA adjusted) | (Intercept) | −0.62 | −1.39 – 0.16 | 0.119 |
|  | Repetition | 1.17 | 0.74 – 1.59 | <0.001*** |
|  | Paradigm | 1.58 | 1.19 – 1.97 | <0.001*** |
|  | Repetition × Paradigm | −0.22 | −0.77 – 0.34 | 0.442 |

+ *p* < 0.1. * *p* < 0.05. ** *p* < 0.01. *** *p* < 0.001.
